# Supplementary material for: A molecular pathology, neurobiology, biochemical, genetic and neuroimaging study of progressive apraxia of speech
Source: Nat Commun. 2021 Jun 8;12:3452. doi: 10.1038/s41467-021-23687-8 (PMC8187627; doi:10.1038/s41467-021-23687-8)
Supplement: Supplementary file 1 — Supplementary Information [file 41467_2021_23687_MOESM1_ESM.pdf]

Supplementary Materials for

A molecular pathology, neurobiology, biochemical, genetic and neuroimaging study of progressive apraxia of speech

Keith A. Josephs, MD, MST, MSc; Joseph R. Duffy, PhD; Heather M. Clark, PhD; Rene L. Utianski, PhD; Edythe A. Strand, PhD; Mary M. Machulda, PhD; Hugo Botha, MD; Peter R. Martin, MS; Nha Trang Thu Pham, BS; Julie Stierwalt, PhD; Farwa Ali, MD; Marina Buciuc, MD, MSc; Matthew Baker, BS; Cristhoper H. Fernandez De Castro, BS; Anthony J. Spychalla, BS; Christopher G. Schwarz, PhD; Robert I Reid, PhD; Matthew L. Senjem, MS; Clifford R. Jack, Jr, MD; Val J. Lowe, MD; Eileen H. Bigio, MD; Ross R. Reichard, MD; Eric. J. Polley, PhD; Nilufer Ertekin-Taner, PhD, MD; Rosa Rademakers, PhD; Michael A. DeTure, PhD; Owen A. Ross, PhD; Dennis W. Dickson, MD; Jennifer L. Whitwell, PhD

*Corresponding author. Email josephs.keith@mayo.edu

This document includes:

Supplementary Text
Supplementary Figs. 1 to 10
Supplementary Tables 1 to 5

Supplementary Text

Statistical details for the neuroimaging models

In the MRI model, volumes were scaled and centered within region to bring measurements from inherently different sized regions to approximately the same magnitude, improving estimation of the single hierarchical model using these scaled volumes. FDG SUVR and DTI FA already were on a comparable scale across regions; raw values were used as the outcome in these modalities and values from the left and right hemisphere were entered into the models.

Algebraically, these three models can equivalently be expressed as:

$$y_{ijk} = \beta_{1k} + \beta_{2k} * time_j + I_{CBD} * (\gamma_{1k} + \gamma_{2k} * time_j) + I_{PSP} * (\gamma_{3k} + \gamma_{4k} * time_j) + \varphi_i$$

where i indicates individual, j indicates timepoint, and k indicates region or tract in the case of the DTI model (simply referred to as region hereafter). y_{ijk} is the outcome (for a given modality) for person i at timepoint j for region k. The terms I_{CBD} and I_{PSP} are indicator functions for whether individual i was given a diagnosis of PSP or CBD, thereby only including the relevant model terms in the estimation at that data point. β_{1k} indicates the region-specific intercept (baseline value) in the entire cohort, β_{2k} the region-specific annual change in the entire cohort, γ_{1k} the region-specific intercept shift for CBD, γ_{2k} the region-specific annual change shift for CBD, γ_{3k} the region-specific intercept shift for PSP, and γ_{4k} the region-specific annual change shift for PSP. φ_i is the person-specific intercept shift, allowing us to use multiple regions-per-scan and multiple scans-per-person in a single modality model.

Of particular interest in these models is comparing γ_{1k} and γ_{2k}, the diagnosis-specific differences at baseline, and comparing γ_{3k} and γ_{4k}, the diagnosis-specific differences in annual change. In addition, the model fits for each diagnosis can be compared at any timepoint to assess whether outcome measures are diverging, converging, or are essentially parallel in these diagnostic groups.

The prior distributions of these parameters were auto scaled to find efficient and appropriate distributions (after internally centering the predictors), the default behavior of the rstanarm software. This means the β_{1k} candidate terms were drawn from independent $N(0, 11.7)$ and β_{2k} candidate terms were drawn from independent $N(0, 4.0)$ distributions in the MRI model, $N(0, 3.1)$ and $N(0, 1.1)$ respectively for the FDG model, and $N(0, 2.0)$ and $N(0, 0.7)$ respectively for the DTI model. Group wise estimation was used for diagnosis specific effects, drawing the γ_{1k} and γ_{3k} parameters simultaneously from, essentially, a bivariate standard normal distribution, allowing for nonzero covariance, in each model. More details can be found in the documentation for the stan_lmer function from the package rstanarm and at this website. Similarly, γ_{2k} and γ_{4k} were drawn from an identical (but independent) bivariate distribution in each model. The prior distribution for the φ_i terms was also, essentially, a standard normal distribution in each model. The overall Sigma, the variability in the outcome measure not described by this model formulation and the analog of the error term in ordinary least squares regression, was drawn from an exponential distribution with rate parameter 1 for the MRI model, 5.3 for the FDG model, and 11 for the DTI model.

Model diagnostics were adequate in all three models. The Monte Carlo standard error of all parameters in all models was approximately zero, the effective sample size of all parameters in all models was in the thousands or tens of thousands except the variance and covariance parameters of the longitudinal group effects in the DTI model, which were above 900. The

mixing parameter, Rhat or $\hat{R}$, was approximately 1 for all parameters in all models. Finally, posterior fits were qualitatively inspected for all regions in all models by comparing model expectations to a scatter plot of the raw data, analogous to the regions selected in Figure 5.The posterior model fits appear to describe these data adequately across regions and modalities when comparing observed and expected outcomes. Results of the hierarchical models were based on 14 Monte Carlo Markov Chains run in parallel, each consisting of 5000 posterior samples.

Statistical details for the clinical trajectory models

To model the change over time in four clinical measures, MDS-UPDRS III, MoCA, WAB-AQ, and ASRS, we fit four mixed effects models, one per clinical test, using clinical score as the outcome predicted by diagnosis-specific intercept and time (years) terms, including diagnosis-specific quadratic terms for time to allow for nonlinearity in the change over the disease course. We also included a random intercept per person, to allow for multiple observations, i.e. multiple clinical visits, per person. In the MDS-UPDRS III and ASRS models, we additionally included a person-specific random effect for linear change which was allowed to be correlated with the person-specific intercept. Convergence issues prevented the inclusion of this person-specific rate term in the MoCA and ASRS models. Time was centered at 5 years from onset of disease to improve estimation in the model.

Statistical methods for correlations between neuroimaging and clinical measures

Spearman rank correlations were calculated within PSP and CBD groups to compare and test for an association between measures of language and AOS and regional metabolism measures. The Western Aphasia Battery Aphasia Quotient and Token Test were compared with the left Broca's and left superior temporal gyrus and the Apraxia of Speech Rating Scale version 3 total score was compared to SMA and Precentral regional metabolism.

Statistical methods for neuroimaging versus AOS subtype

Consistent with our overall neuroimaging approach, the cortical regions (SMA and precentral/motor cortex) were assessed using FDG-PET SUVR, and the subcortical structures (striatum, globus pallidus, subthalamic nucleus and substantia nigra) were assessed using MRI volumes. Scans closest to death were utilized. In order to account for confounding effects of age in brain volume, MRI volumes were converted to age- and total intracranial volume (TIV)-corrected Z scores using a model predicting volume by age and TIV in 36 cognitively normal controls. Specifically, to calculate these Z scored volumes, we used the model fit in controls to predict expected volume for each case based on age at scan and TIV. Then we subtracted the expected volume from the observed volume and divided this difference by the standard deviation of the residuals from the original model fit. We then performed non-parametric Wilcoxon Rank Sum tests using regional imaging measures to compare regional metabolism and age- and TIV-adjusted volumes between phonetic and prosodic subtypes of AOS.

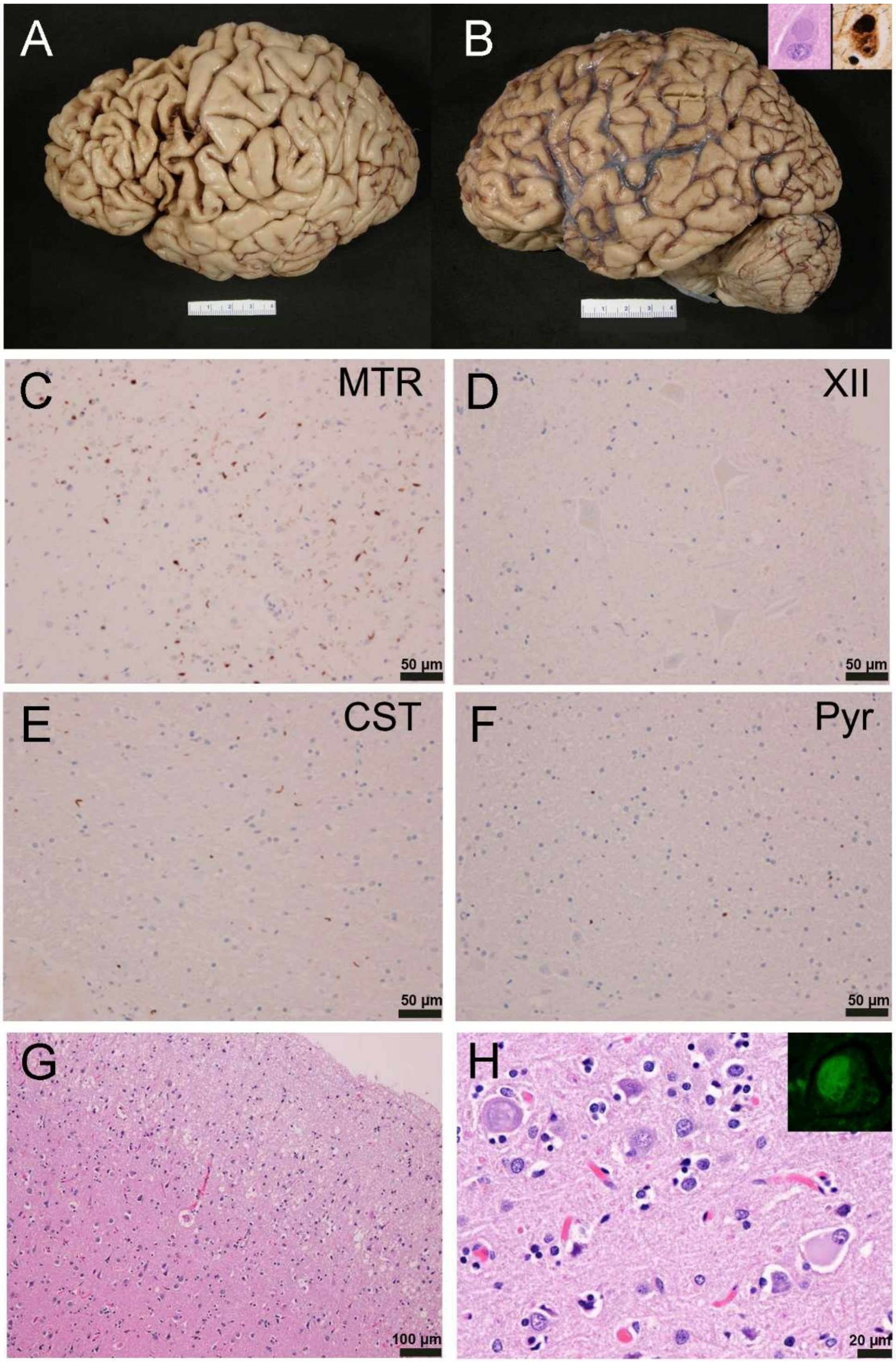

Supplementary Fig. 1. Gross and histological novel findings in progressive apraxia of speech (PAOS) cases. Gross brain pictures of the two +AOS cases with Picks disease (A and B) including a novel case of atypical Picks disease without lobar atrophy and Pick bodies (B). Scales in panel A and B represent cm. One of the PAOS-progressive supranuclear palsy (PSP) cases showed features diagnostic of PSP with corticospinal tract degeneration including tau-

positive inclusions in motor cortex (C), corticospinal tract (E) and medullary pyramid (F) without loss of motor neurons or inclusions in the motor neurons of cranial nerve XII (D). Another PAOS-PSP case showed severe neuronal loss in the motor cortex (G) with some remaining Betz cells having tau-immunoreactive globose neurofibrillary tangles (H). Tau-immunohistochemistry B (inset), (C, E, F) and H (inset), TDP-43 immunohistochemistry (D) and Hematoxylin and Eosin (G and H). The images shown do not represent experiments themselves but identifiable lesions which were observed at the time of original analysis by one investigator (DWD) and were assessed with digital images via zoom with two investigators (DWD and KAJ) at a second time-point.

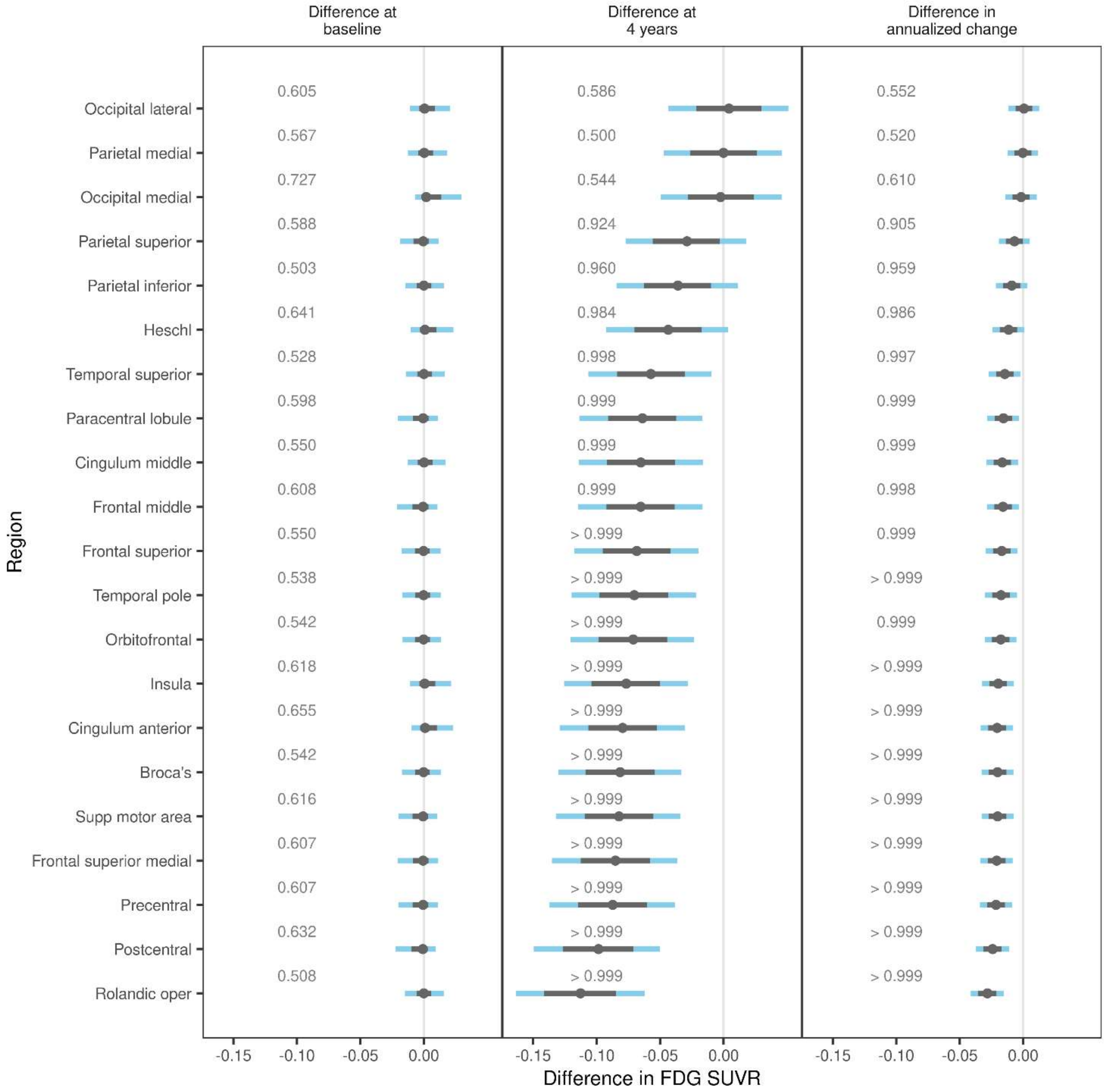

Supplementary Fig. 2. FDG-PET comparisons between progressive apraxia of speech (PAOS) patients with corticobasal degeneration (CBD) or progressive supranuclear palsy (PSP) pathology. Estimated differences in FDG standard uptake value ratio (SUVR) at baseline (left panel), 4 years from baseline (middle panel) and in annualized change of FDG SUVR (right panel) between PAOS-CBD and PAOS-PSP are shown. The estimated differences are the PAOS-CBD estimate minus PAOS-PSP estimate, with posterior probability of a difference greater or less than zero printed slightly above each row in grey text. The estimates to the right of the zero line in each pane indicate higher FDG SUVR in PAOS-CBD than PAOS-PSP at baseline or 4-years from baseline, or in the case of the third panel, slower annualized change in PAOS-CBD than PAOS-PSP. Estimates to the left of the line indicate a reversal with PAOS-PSP having higher FDG SUVR or slower annualized change in these regions. Within each row, the

point estimate is a grey dot, surrounded by an 80% posterior interval (showing probability ≥ 90% if not crossing the zero line) in grey and a 98% posterior interval (showing probability ≥ 99% if not crossing the zero line) in light blue. Source data are provided as a Source Data file.

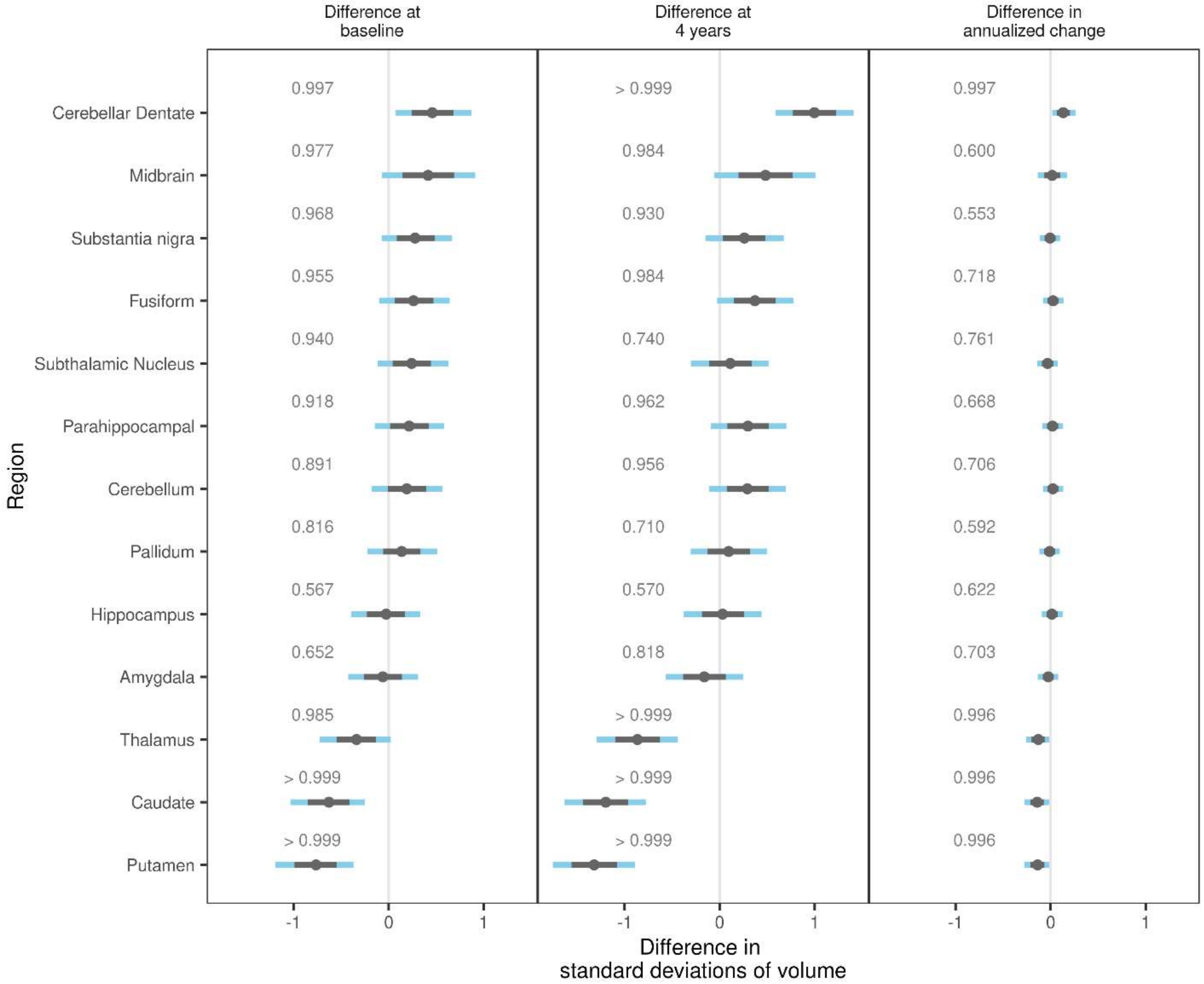

Supplementary Fig. 3. MRI volume comparisons between progressive apraxia of speech (PAOS) patients with corticobasal degeneration (CBD) or progressive supranuclear palsy (PSP) pathology. Estimated differences in volume at baseline (left panel), 4-years from baseline (middle panel) and in annualized change of volume (right panel) between PAOS-CBD and PAOS-PSP are shown. The estimated differences are the PAOS-CBD estimate minus PAOS-PSP estimate, with posterior probability of a difference greater or less than zero printed slightly above each row in grey text. The estimates to the right of the zero line in each pane indicate higher volume in PAOS-CBD than PAOS-PSP at baseline or 4-years from baseline, or in the case of the third panel, slower annualized change in PAOS-CBD than PAOS-PSP. Estimates to the left of the line indicate a reversal with PAOS-PSP having higher volume or slower annualized change in these regions. Within each row, the point estimate is a grey dot, surrounded by an 80% posterior interval (showing probability ≥ 90% if not crossing the zero line) in grey and a 98% posterior interval (showing probability ≥ 99% if not crossing the zero line) in light blue. Source data are provided as a Source Data file.

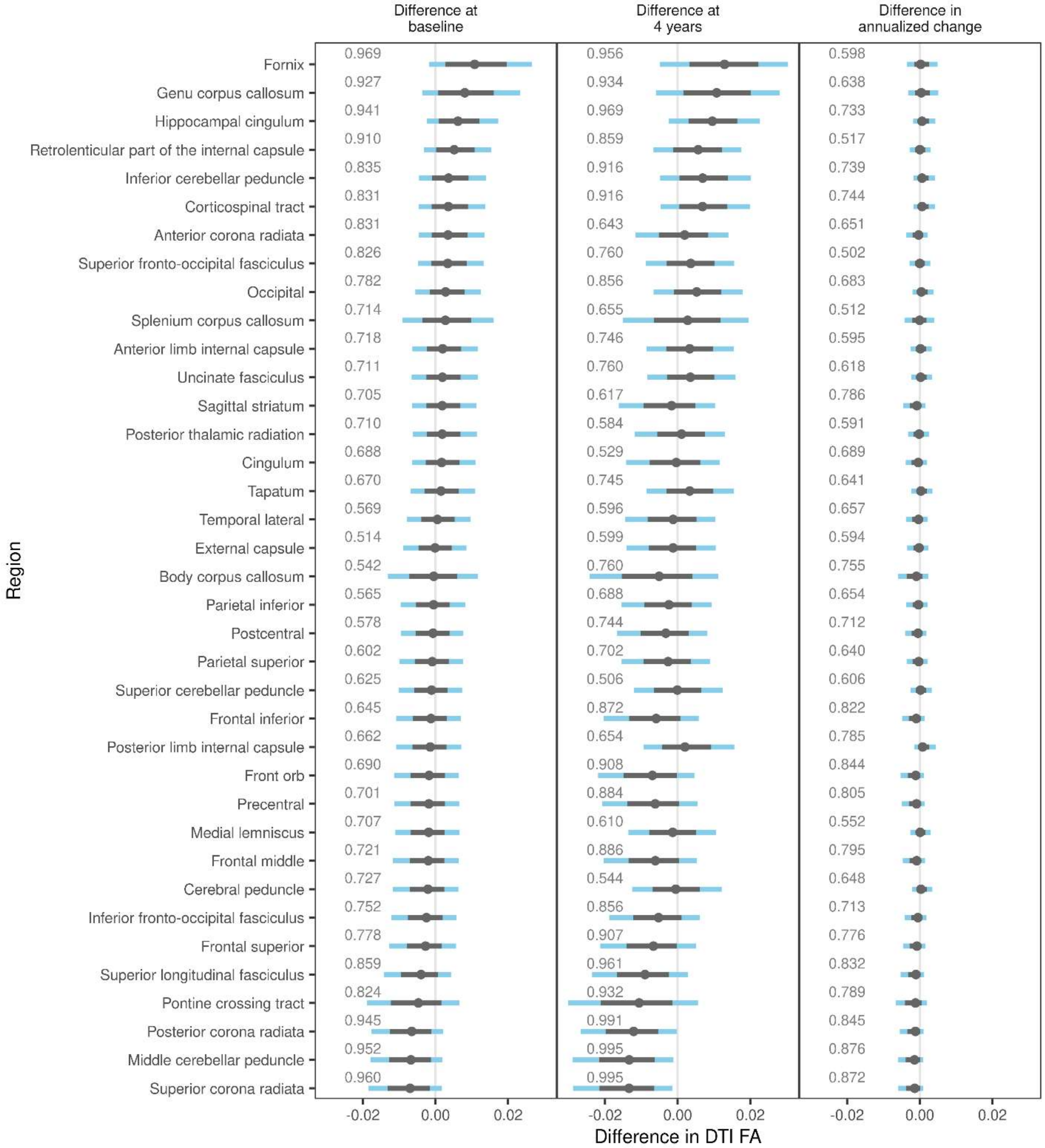

Supplementary Fig. 4. Diffusion tensor imaging (DTI) fractional anisotropy (FA) comparisons between progressive apraxia of speech (PAOS) patients with corticobasal degeneration (CBD) or progressive supranuclear palsy (PSP) pathology. Estimated differences in DTI FA at baseline (left panel), 4-years from baseline (middle panel) and in annualized change of DTI FA (right panel) between PAOS-CBD and PAOS-PSP from the anchored-at-baseline model are shown. The estimated differences are the PAOS-CBD estimate minus PAOS-PSP estimate, with posterior probability of a difference greater or less than zero (whichever is greater) printed slightly above each row in grey text. The estimates to the right of the zero line in each pane indicate higher DTI FA in PAOS-CBD than PAOS-PSP at baseline or

4-years from baseline, or in the case of the third panel, slower annualized change in PAOS-CBD than PAOS-PSP. Estimates to the left of the line indicate a reversal with PAOS-PSP having higher DTI FA or slower annualized change in these regions. Within each row, the point estimate is a grey dot, surrounded by an 80% posterior interval (showing probability ≥ 90% if not crossing the zero line) in grey and a 98% posterior interval (showing probability ≥ 99% if not crossing the zero line) in light blue. Source data are provided as a Source Data file.

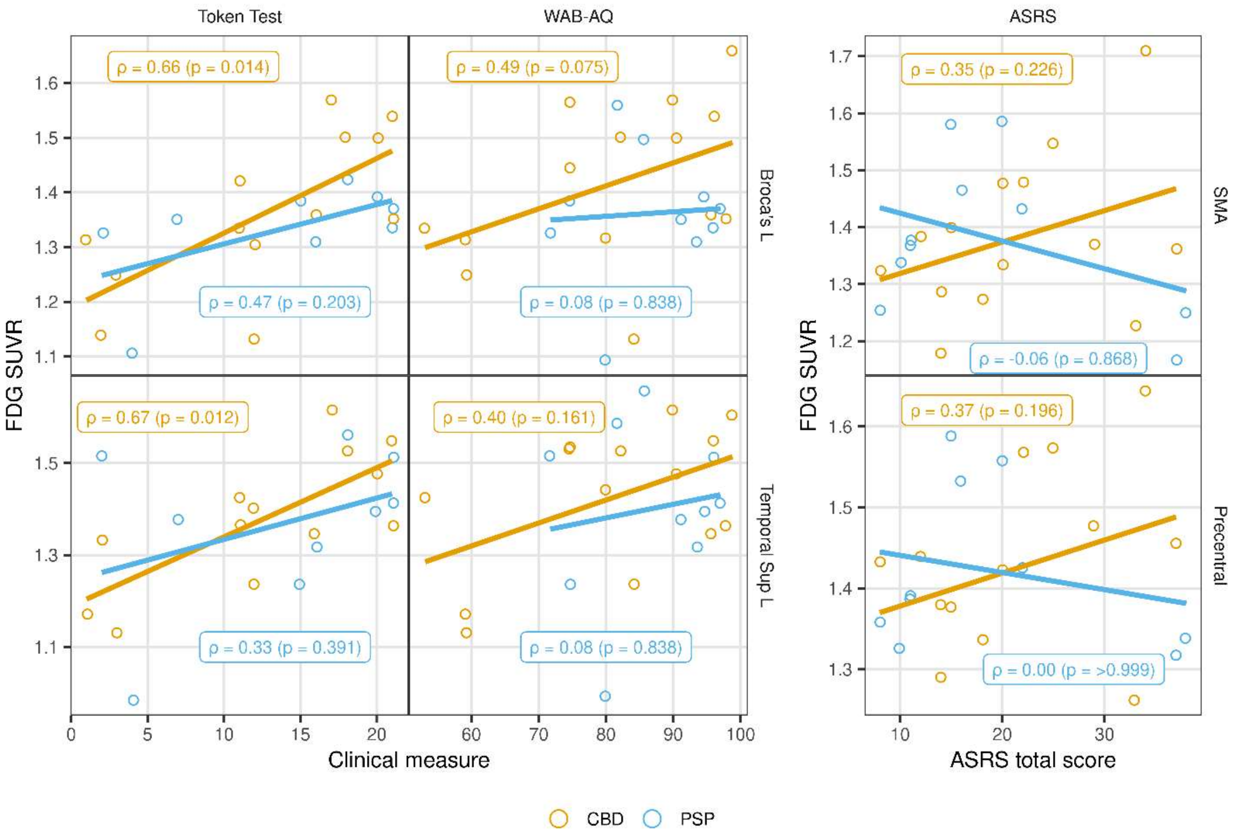

Supplementary Fig. 5. **Scatterplots showing the relationship between clinical measures and FDG-PET metabolism.** The Token Test and Western Aphasia Battery Aphasia Quotient (WAB-AQ) were included as measures of language ability and were related to the language areas of Broca's area and left superior temporal gyrus. The Apraxia of Speech Rating Scale (ASRS) was included as a measure of apraxia of speech severity and was assessed in relation to the supplementary motor area (SMA) and precentral cortex; two cortical regions that are commonly abnormal in progressive apraxia of speech. The last available visit was used for all patients for each test. Spearman correlations were assessed separately for progressive apraxia of speech patients with corticobasal degeneration (CBD) and progressive supranuclear palsy (PSP), with CBD cases, trend-lines and p-values shown in orange, and PSP shown in blue. Spearman rho correlations were calculated using a two-sided test via an asymptotic t distribution approximation. Source data are provided as a Source Data file.

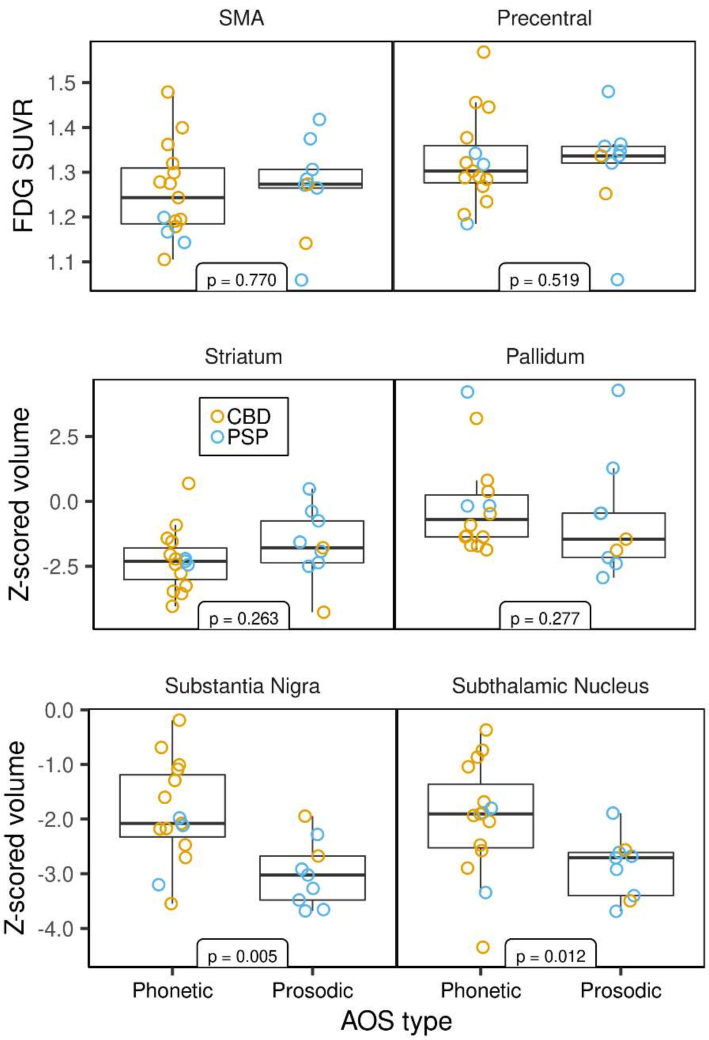

Supplementary Fig. 6. Neuroimaging comparisons across phonetic and prosodic apraxia of speech (AOS) targeting the corticostriatal and pallidonigraluysian networks. Cortical regions were assessed using FDG-PET standard uptake value ratios (SUVRs) and subcortical structures were assessed using age- and total intracranial volume-corrected Z scores of MRI volume. The last available scan was used for all patients. The corticobasal degeneration (CBD) and progressive supranuclear palsy (PSP) patients are shown in orange and blue respectively. SMA = supplementary motor area. N=15 phonetic cases and N=9 prosodic cases. Boxes represent lower quartile, median and upper quartile, with whiskers extending to the farthest point at most 1.5*inter-quartile range from each quartile. P values are from two sample Wilcoxon rank-sum test. One CBD phonetic case with z-scored volume of 13.2 in the pallidum was excluded from the pallidum plot to maintain readability of the y-axis. Source data are provided as a Source Data file.

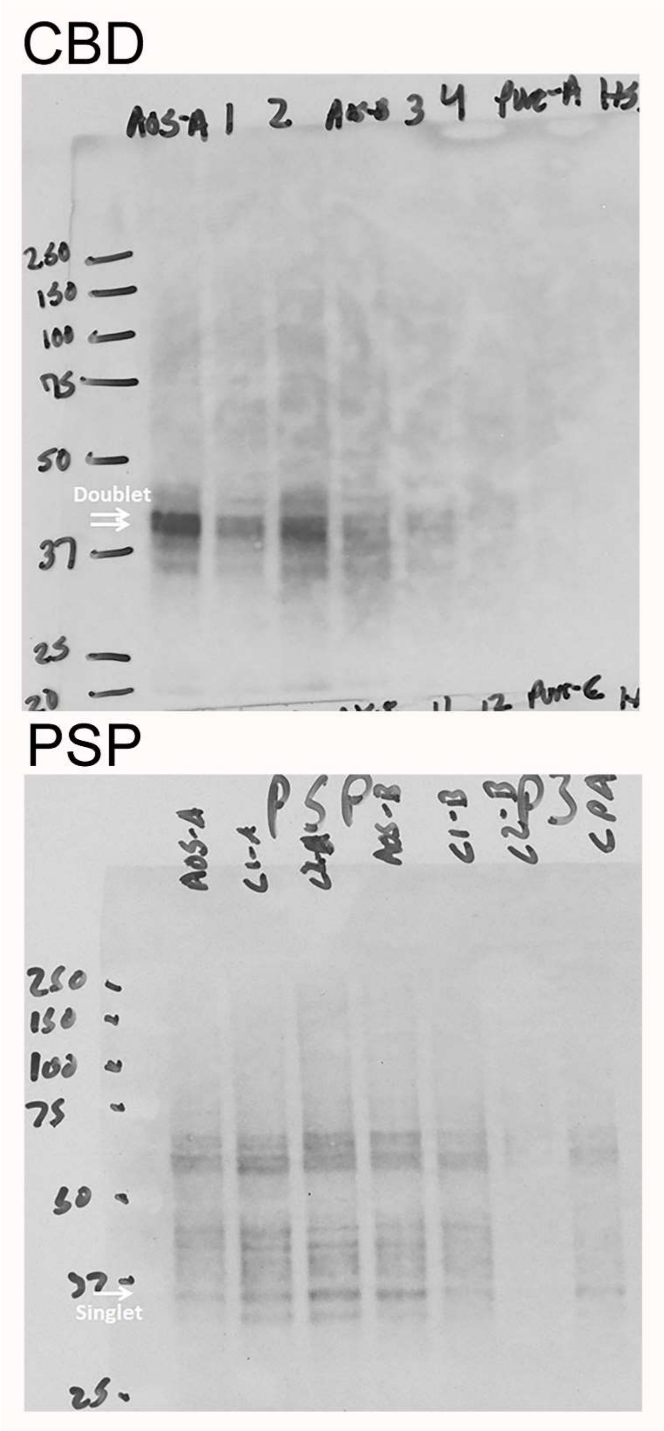

Supplementary Fig. 7. Full scan western blots showing verification by reprobing the CBD and PSP blots with PHF1. Top panel shows representative corticobasal degeneration (CBD) cases and bottom panel shows representative progressive supranuclear palsy (PSP) cases. Apraxia of speech (AOS) denotes the progressive apraxia of speech cases; 1-4 denotes the matched controls; and Pure-A denotes pure control with no co-pathologies.

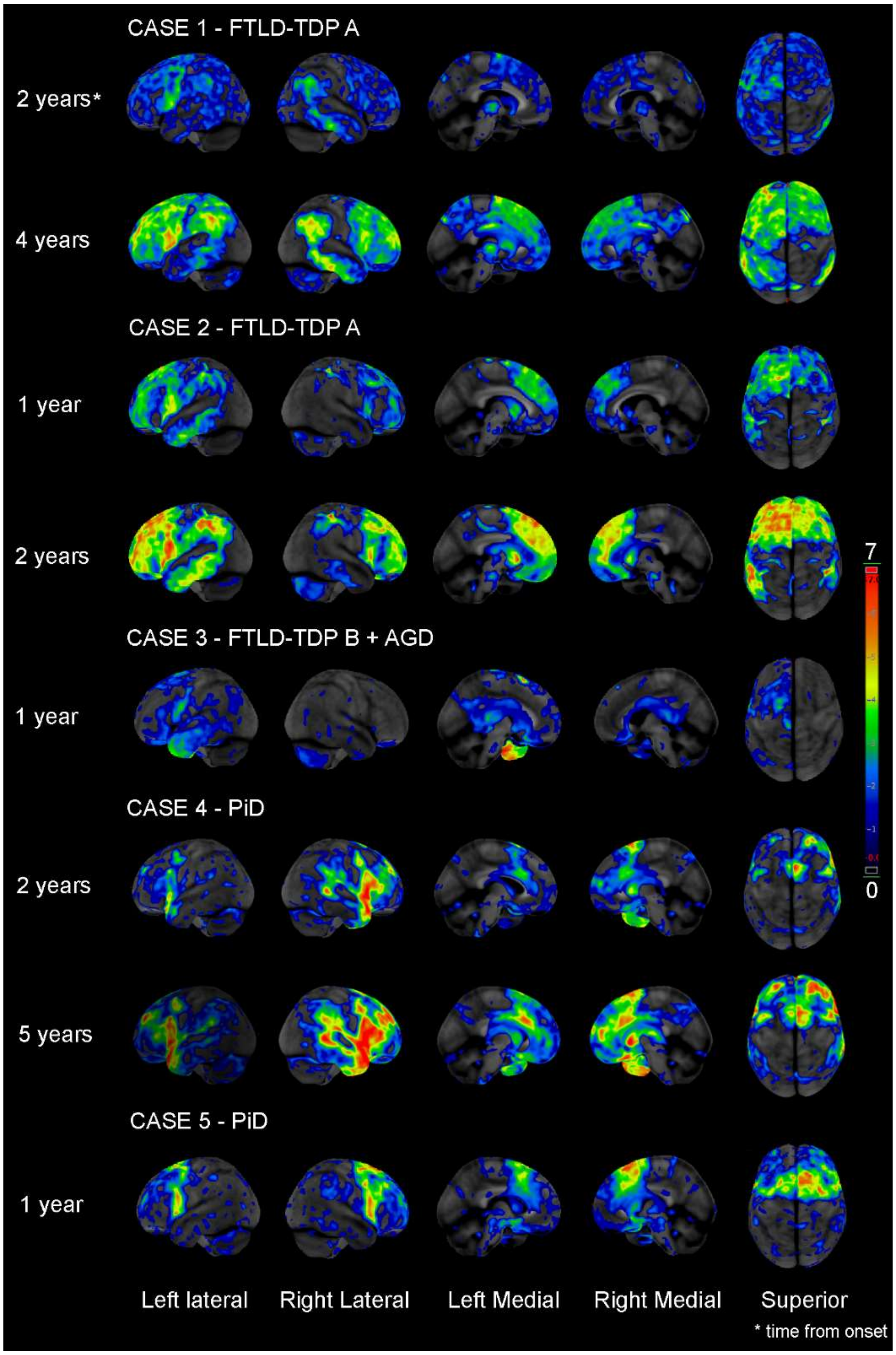

Supplementary Fig. 8. FDG-PET scans for the +AOS patients. Cortex ID Z score maps are shown. Three patients had longitudinal FDG-PET. Years from disease onset for each FDG-PET are shown on the left. AGD = argyrophilic grains disease; PiD = Pick's disease

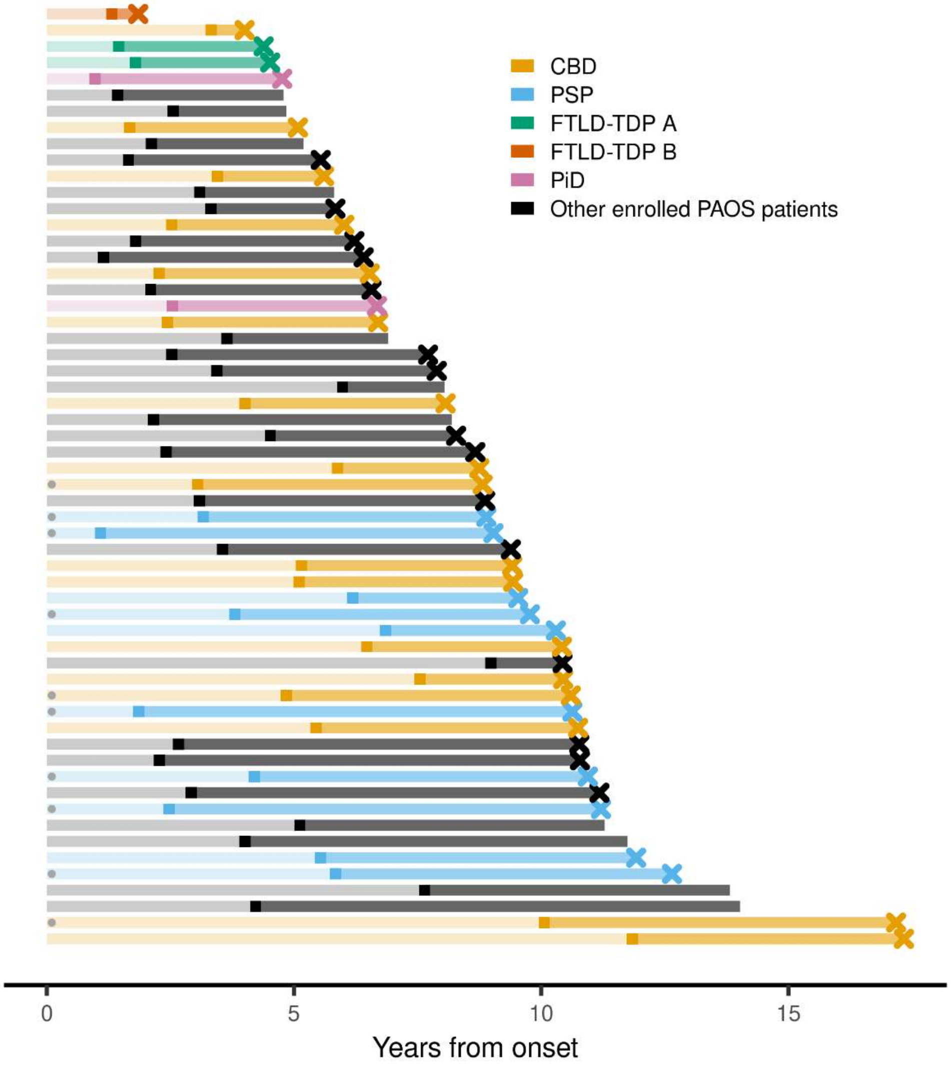

Supplementary Fig. 9. Swim plot illustrating follow-up for all PAOS patients enrolled into a longitudinal NIH-funded grant. Each horizontal segment represents an individual in this plot. The CBD, PSP, FTLD-TDP and PiD patients that have died, undergone autopsy and are included in the current study are highlighted. The time of enrolment and first visit in the research grant is the dark square in each segment. Please note earlier clinical visits may have occurred. Left of the dark point represents the time from onset to first research visit, and right of the point represents follow up from first research visit to date of last contact. An × at the end of a segment indicates the patient has died. A grey dot in the horizontal segment highlights those patients that were used in the original description of AOS types in 2013 who have now died. Source data are provided as a Source Data file.

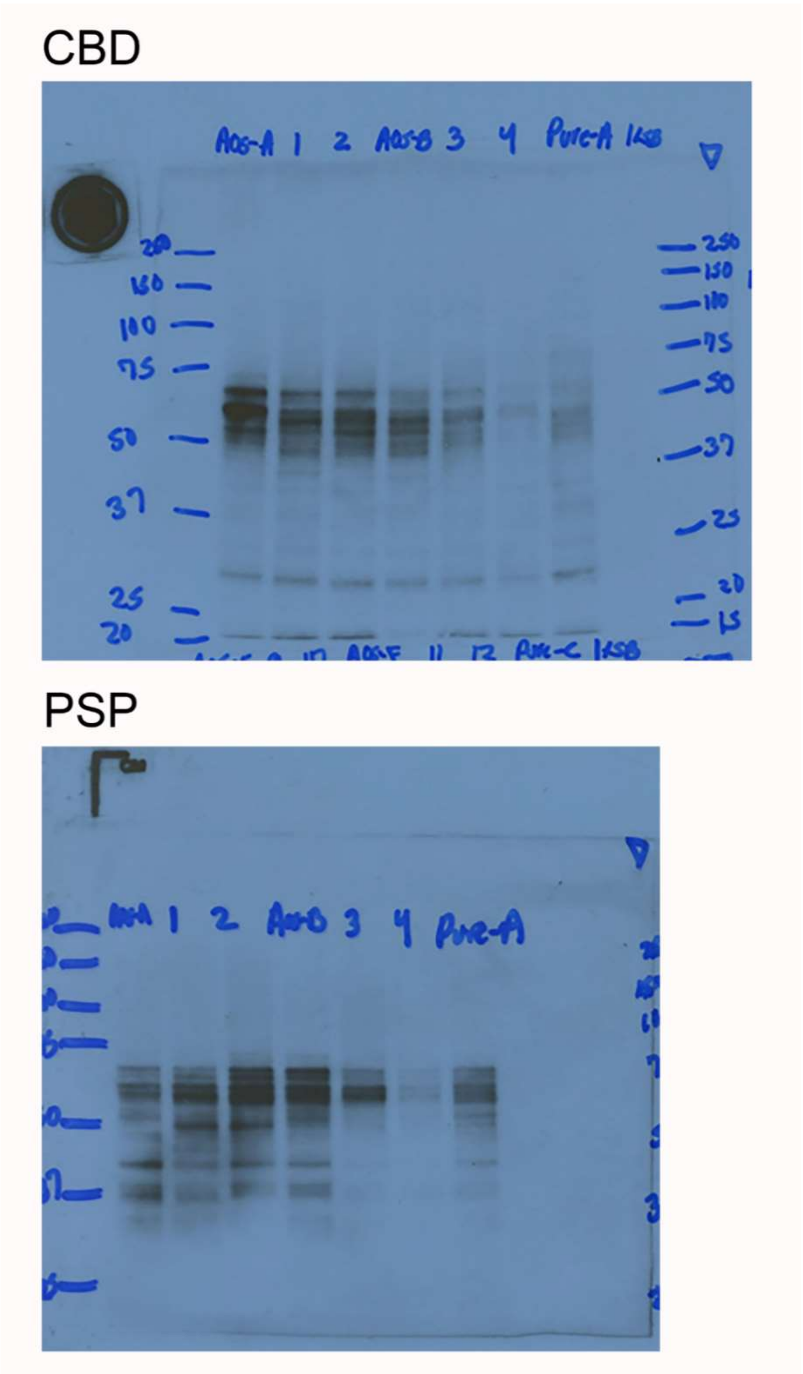

Supplementary Fig. 10: Full scan images of the tau biochemistry western blots. Western blot findings in representative corticobasal degeneration (CBD) (top panel) and progressive supranuclear palsy (PSP) (bottom panel) cases. Apraxia of speech (AOS) denotes the progressive apraxia of speech cases; 1-4 denotes the matched controls; and Pure-A denotes pure controls with no co-pathologies. This experiment was replicated independently in eight CBD cases and seven PSP cases with similar results. Plots also provided in source file.

	PPAOS		AOS-PAA		+AOS			Total N=32
	**CBD N=9**	**PSP N=6**	**CBD N=8**	**PSP N=4**	**FTLD-TDP A N=2**	**FTLD-TDP B* N=1**	**PiD N=2**	
Thal phase	0 (0, 2)	2 (0, 2)	0 (0, 1.0)	0 (0, 2)	0, 3	0	0	0 (0, 2)
Braak stage	2 (2, 3)	3 (3, 3)	2 (1, 3)	3.5 (2, 4)	1, 3	3	0, 2	2 (2, 3)
PART, N %	4 (44%)	2 (33%)	4 (50%)	1 (25%)	0	1	1 (50%)	13 (41%)
ARTAG, N %	7 (78%)	3 (50%)	3 (38%)	2 (50%)	0	0	0	15 (47%)
AGD, N %	4 (44%)	2 (33%)	8 (100%)	2 (50%)	0	0	0	16 (50%)
VD, N %	0	3 (50%)	0	1 (25%)	0	0	0	4 (13%)
LBD, N %	0	0	0	0	0	0	0	0
TDP-43	0	0	0	0	2	1	0	3

Supplementary Table 1. Additional co-pathologies in all 32 PAOS patients

ARTAG = Age-related tau astrogliopathy; LBD = Lewy body disease; PART = Primary age-related tauopathy; TDP = TAR DNA binding protein; VD = Vascular disease. * Case also had focal argyrophilic grains disease (AGD) in limbic regions. Data shown as median (inter-quartile range) for cells with N≥3 or N (%). Source data are provided as a Source Data file.

	Phonetic		Prosodic	
**Age Onset**	**CBD**	**PSP**	**CBD**	**PSP**
60	84%	16%	48%	52%
61	82%	18%	45%	55%
62	80%	20%	42%	58%
63	78%	22%	39%	61%
64	76%	24%	36%	64%
65	74%	26%	34%	66%
66	71%	29%	31%	69%
67	69%	31%	28%	72%
68	66%	34%	26%	74%
69	63%	37%	23%	77%
70	60%	40%	21%	79%
71	57%	43%	19%	81%
72	54%	46%	17%	83%
73	51%	49%	16%	84%
74	48%	52%	14%	86%
75	45%	55%	13%	87%
76	42%	58%	11%	89%
77	39%	61%	10%	90%
78	36%	64%	9%	91%
79	33%	67%	8%	92%
80	30%	70%	7%	93%

Supplementary Table 2. Predicted probabilities of CBD versus PSP pathology in PAOS patients at different onset ages and whether the AOS is the phonetic or prosodic AOS subtype. Probabilities were calculated from a logistic regression model predicting pathology by age and AOS type. Source data are provided as a Source Data file.

Clinical model	Model term	Estimate	p value
MoCA	Intercept	24.08	<0.001
	time	-1.13	0.004
	$time^2$	0.02	0.595
	PSP	1.67	0.501
	time:PSP	0.55	0.324
	$time^2$:PSP	-0.05	0.389
MDS-UPDRS III	Intercept	20.49	<0.001
	time	4.29	0.014
	$time^2$	0.39	<0.001
	PSP	-3.87	0.588
	time:PSP	-1.39	0.574
	$time^2$:PSP	-0.02	0.853
WAB-AQ	Intercept	87.24	<0.001
	time	-3.70	<0.001
	$time^2$	0.09	0.298
	PSP	5.97	0.370
	time:PSP	2.59	0.018
	$time^2$:PSP	-0.07	0.569
ASRS	Intercept	20.79	<0.001
	time	1.90	<0.001
	$time^2$	-0.08	0.326
	PSP	-2.13	0.441
	time:PSP	-0.14	0.827
	$time^2$:PSP	0.16	0.090

Supplementary Table 3. Model estimates and p values for the clinical trajectory analyses
The intercept, time and $time^2$ terms explain the model fit, with time referring to the linear fit and $time^2$ referring to the non-linear fit. The time:PSP term refers to differences between PSP and CBD in the linear term, while $time^2$:PSP refers to differences between PSP and CBD in the non-linear term. Separate models were run for the Montreal Cognitive Assessment Battery (MoCA), Movement Disorders Society sponsored revision of the Unified Parkinson's Disease Rating Scale part III (MDS-UPDRS III), Western Aphasia Battery Aphasia Quotient (WAB-AQ), and Apraxia of Speech Rating Scale (ASRS). Model p values are from a t-test using Satterthwaite's degrees of freedom method to estimate the degrees of freedom of each term. Source data are provided as a Source Data file.

Region	TDP-43 lesion count	AGD lesion count
Superior Frontal	2+	0
Peri-Rolandic	1+	0
Superior Temporal	2+	0
Inferior Parietal	0	0
Cingulate	2+	0
Basal nucleus of Meynert	1+	2+
Amygdala	2+	2+
Entorhinal cortex	2+	2+
Hippocampus CA1	1+	2+
Hippocampus dentate	1+	0
Basal ganglia	0	0
Midbrain Tegmentum	0	0
Medullary Tegmentum	0	0
Medullary CN XII	1+	0
Medullary Inferior Olive	0	0

Supplementary Table 4. Semi-quantitation of TDP-43 and argyrophilic grains disease (AGD) pathology in +AOS Case 3. Scale: none=0; scant number of inclusions =1+, moderate number of inclusions = 2+, frequent number of inclusions = 3+. TDP-43 lesion count based on neuronal cytoplasmic inclusions. TDP-43 immunohistochemistry based on phospho-TDP antibody. AGD based on tau immunohistochemistry.

Primer name	Primer sequence
MAPT 1F	CACGACGTTGTAAAACGACCTGAGATCTGCCTGCCATG
MAPT 1R	GGATAACAATTTCACACAGGGTGTCTGGCCATTATCTCACTG
MAPT 7F	CACGACGTTGTAAAACGACTAGGAGGCAAAGGGTCAC
MAPT 7R	GGATAACAATTTCACACAGGTTCATTCTCAGTGGCCTAAG
MAPT 9F	CACGACGTTGTAAAACGACAGTGGTGAGCCTGGGAATG
MAPT 9R	GGATAACAATTTCACACAGGATGCACAGTCCCACGACTC
MAPT 10F	CACGACGTTGTAAAACGACTGCCTCTGCCAAGTCCGAAAG
MAPT 10R	GGATAACAATTTCACACAGGCCAGATCCTGAGAGCCCAAGAAG
MAPT 11F	CACGACGTTGTAAAACGACTTGGCAGAATTTCGACAACAC
MAPT 11R	GGATAACAATTTCACACAGGAGCAGTTCCAGCCTCACCAG
MAPT 12F	CACGACGTTGTAAAACGACGTCCTGTCATTGTCTTCTTC
MAPT 12R	GGATAACAATTTCACACAGGACCCACTGGATGCTGCTGAG
MAPT 13F	CACGACGTTGTAAAACGACGCGATAGAGCAAGACCCTG
MAPT 13R	GGATAACAATTTCACACAGGTTAACCGAACTGCGAGGAG
GRN EX0F	CACGACGTTGTAAAACGACCGCCTGCAGGATGGGTTAAGG
GRN EX0R	GGATAACAATTTCACACAGGGCGTCACTGCATTACTGCTTCC
GRN EX1F	CACGACGTTGTAAAACGACGGGCTAGGGTACTGAGTGAC
GRN EX1R	GGATAACAATTTCACACAGGAGTGTTGTGGGCCATTTG
GRN EX2F	CACGACGTTGTAAAACGACTGCCCAGATGGTCAGTTC
GRN EX2R	GGATAACAATTTCACACAGGGCTGCACCTGATCTTTGG
GRN EX3F	CACGACGTTGTAAAACGACGGCCACTCCTGCATCTTTAC
GRN EX3R	GGATAACAATTTCACACAGGTGAATGAGGGCACAAGGG
GRN EX4F	CACGACGTTGTAAAACGACGCCTTAGTGTCACCCTCAAAC
GRN EX4R	GGATAACAATTTCACACAGGAGTGCACCCTGTCTTCACAG
GRN EX4F SEQ	CTTCCCTGAGTGGGCTGG
GRN EX5F	CACGACGTTGTAAAACGACGTTATGGTCGATGGCTCCTG
GRN EX5R	GGATAACAATTTCACACAGGATGACCGAGCTGGACAAGG
GRN EX6F	CACGACGTTGTAAAACGACGGGCCTCATTGACTCCAAGTGTA
GRN EX6R	GGATAACAATTTCACACAGGGGTCTTTGTCACTTCCAGGCTCA
GRN EX7F	CACGACGTTGTAAAACGACTCCCTGTGTGCTACTGAG
GRN EX7R	GGATAACAATTTCACACAGGAAGCAGAGAGGACAGGTC
GRN EX8F	CACGACGTTGTAAAACGACTACCCTCCATCTTCAACAC
GRN EX8R	GGATAACAATTTCACACAGGTCACAGCACACAGCCTAG
GRN EX9F	CACGACGTTGTAAAACGACATACCTGCTGCCGTCTAC
GRN EX9R	GGATAACAATTTCACACAGGGAGGGCAGAAAGCAATAG
GRN EX10F	CACGACGTTGTAAAACGACTGTCCAATCCCAGAGGTATATG
GRN EX10R	GGATAACAATTTCACACAGGACGTTGCAGGTGTAGCCAG
GRN EX11F	CACGACGTTGTAAAACGACAGACATCGGCTGTGACCAG
GRN EX11R	GGATAACAATTTCACACAGGGCCGATCAGCACAACAGAC
GRN EX12F	CACGACGTTGTAAAACGACCATGATAACCAGACCTGC
GRN EX12R	GGATAACAATTTCACACAGGAGGGAGAATTTGGTTAGG
Chr9 FAM assay F	FAM-CAAGGAGGGAAACAACCGCAGCC

Chr9 FAM assay R	GCAGGCACCGCAACCGCAG
Chr9 3primer F Flu	FAM-TGTAAAACGACGGCCAGTCAAGGAGGGAAACAACCGCAGCC
Chr9 3primer R	CAGGAAACAGCTATGACCGGGCCCGCCCCGACCACGCCCCGGCCCCGGCCCCGG
Chr9 3primer M13R	CAGGAAACAGCTATGACC

Supplementary Table 5. Primer sequences for *GRN* and *MAPT* sequencing, and for the C9 screening
