# Supplementary material for: A molecular pathology, neurobiology, biochemical, genetic and neuroimaging study of progressive apraxia of speech
Source: Nat Commun. 2021 Jun 8;12:3452. doi: 10.1038/s41467-021-23687-8 (PMC8187627; doi:10.1038/s41467-021-23687-8)
Supplement: Supplementary file 2 — Reporting Summary [file 41467_2021_23687_MOESM2_ESM.pdf]

nature research

Corresponding author(s): Keith A. Josephs

Last updated by author(s): May 7, 2021

Reporting Summary

Nature Research wishes to improve the reproducibility of the work that we publish. This form provides structure for consistency and transparency in reporting. For further information on Nature Research policies, see our Editorial Policies and the Editorial Policy Checklist.

Statistics

For all statistical analyses, confirm that the following items are present in the figure legend, table legend, main text, or Methods section.

n/a	Confirmed	
☐	☒	The exact sample size (*n*) for each experimental group/condition, given as a discrete number and unit of measurement
☐	☒	A statement on whether measurements were taken from distinct samples or whether the same sample was measured repeatedly
☐	☒	The statistical test(s) used AND whether they are one- or two-sided<br>*Only common tests should be described solely by name; describe more complex techniques in the Methods section.*
☐	☒	A description of all covariates tested
☐	☒	A description of any assumptions or corrections, such as tests of normality and adjustment for multiple comparisons
☐	☒	A full description of the statistical parameters including central tendency (e.g. means) or other basic estimates (e.g. regression coefficient) AND variation (e.g. standard deviation) or associated estimates of uncertainty (e.g. confidence intervals)
☐	☒	For null hypothesis testing, the test statistic (e.g. *F*, *t*, *r*) with confidence intervals, effect sizes, degrees of freedom and *P* value noted<br>*Give P values as exact values whenever suitable.*
☐	☒	For Bayesian analysis, information on the choice of priors and Markov chain Monte Carlo settings
☐	☒	For hierarchical and complex designs, identification of the appropriate level for tests and full reporting of outcomes
☐	☒	Estimates of effect sizes (e.g. Cohen's *d*, Pearson's *r*), indicating how they were calculated

Our web collection on statistics for biologists contains articles on many of the points above.

Software and code

Policy information about availability of computer code

Data collection	RedCap 10.0.28; Microsoft Access 15.0
Data analysis	SPM12 (revision 8369) with default settings (https://www.fil.ion.ucl.ac.uk/spm/); Advanced Registration Tools (ANTs) version 1.9.x (https://github.com/ANTsX/ANTs) ; MCALT v1.4 (https://www.nitrc.org/projects/mcalt/); CortexID suite 2.1 (GE Healthcare, Waukesha, Wisconsin); statistical software R (https://www.R-project.org/) version 3.6.2 in conjunction with the rstanarm package version 2.21.1 running STAN version 2.21.0 (https://mc-stan.org/rstanarm); lme4 package version 1.1-25; QuantStudio Real-Time PCR Software version 6 (Thermo Fisher Scientific, Waltham, MA)

For manuscripts utilizing custom algorithms or software that are central to the research but not yet described in published literature, software must be made available to editors and reviewers. We strongly encourage code deposition in a community repository (e.g. GitHub). See the Nature Research guidelines for submitting code & software for further information.

Data

Policy information about availability of data

All manuscripts must include a data availability statement. This statement should provide the following information, where applicable:

- Accession codes, unique identifiers, or web links for publicly available datasets
- A list of figures that have associated raw data
- A description of any restrictions on data availability

The data that support the findings of this study are available from the corresponding author upon reasonable request.

Field-specific reporting

Please select the one below that is the best fit for your research. If you are not sure, read the appropriate sections before making your selection.

- [x] Life sciences
- [ ] Behavioural & social sciences
- [ ] Ecological, evolutionary & environmental sciences

For a reference copy of the document with all sections, see nature.com/documents/nr-reporting-summary-flat.pdf

Life sciences study design

All studies must disclose on these points even when the disclosure is negative.

Sample size	We recruited all patients that had been recruited over 10 years that subsequently died and had a brain autopsy.
Data exclusions	No data was excluded
Replication	Replication of the hierarchical models were based on 14 Monte Carlo Markov Chains run in parallel, each consisting of 5000 posterior samples. Elastic-net regularization was also used which shrinks effect estimates to more generalizable levels, decreases overfitting, and increases the likelihood results can be replicated. The semi-quantitative regional lesion counts were performed at the time of original histological analysis and independently repeated months to years later for each case at a second time-point by the same investigator (DWD).
Randomization	Participants were allocated to groups based on their clinical diagnoses (including apraxia of speech subtype) during life and their molecular pathology at autopsy.
Blinding	The investigators were blinded to group allocation during data collection and analysis

Reporting for specific materials, systems and methods

We require information from authors about some types of materials, experimental systems and methods used in many studies. Here, indicate whether each material, system or method listed is relevant to your study. If you are not sure if a list item applies to your research, read the appropriate section before selecting a response.

Materials & experimental systems

n/a	Involved in the study	
[ ]	[x]	Antibodies
[x]	[ ]	Eukaryotic cell lines
[x]	[ ]	Palaeontology and archaeology
[x]	[ ]	Animals and other organisms
[ ]	[x]	Human research participants
[ ]	[x]	Clinical data
[x]	[ ]	Dual use research of concern

Methods

n/a	Involved in the study	
[x]	[ ]	ChIP-seq
[x]	[ ]	Flow cytometry
[ ]	[x]	MRI-based neuroimaging

Antibodies

Antibodies used	Antibody, Supplier, Catalog #, Dilution, are all shown below:<br>CP13; Peter Davies gift; n/a; 1:1000<br>pS409/410; Cosmo Bio Co., LTD; CAC-TIP-PTD-P01; 1:5,000<br>6F/3D; DAKO (Carpinteria, CA); M0872; 1:250<br>RD3; Millipore (Temecula, CA); C05-803; 1:5000<br>NACP; Mayo Clinic; n/a; 1:3,000<br>E1; Leonard Petrucelli (Mayo Clinic, Jacksonville, FL); n/a; 1:1000<br>RD4; Millipore (Temecula, CA); 05-804; 1:5000<br>PHF1; Peter Davies gift; n/a/; 1:500
Validation	Antibody<br>- Supplier<br>- Catalog #<br>- Validation<br>- RRID<br>- Reference<br><br>CP13<br>- Peter Davies gift

- n/a
- Validation not found in original reference
- AB_2314223
- Jicha et al., J Neurosci, 1999
Herkovits et al., Neurobiol Dis, 2006
https://www.alzforum.org/antibodies/tau-phos-ser202-cp13

pS409/410
- Cosmo Bio Co., LTD
- CAC-TIP-PTD-P01
- specificity was verified by enzyme-linked immunosorbent assay and immunoblot.
- Hasegawa et al. Annals of Neurology 2008; 64(1): 60-70

6F/3D
- DAKO (Carpinteria, CA)
- M0872
- validation not found on DAKO website. 6F/3D was shown to react with beta-amyloid peptides corresponding to residues 9-14.
- Matsunaga et al. Biochem. 2002; 361: 547-556

RD3
- Millipore (Temecula, CA)
- C05-803
- specificity of RD3 was determined with immunohistochemistry in the following tauopathies: Pick's disease, progressive supranuclear palsy and Alzheimer's disease. Biochemical confirmation of specificity utilized western blots of these three diseases, as well as recombinant tau proteins. RD3 stained inclusions in Pick's disease but not progressive supranuclear palsy. In Alzheimer's disease, neurofibrillary tangles tangles were immunolabeled with RD3.
- Togo et al. J Neuropathology and Experimental Neurology, 2002; 61(6):547-556

NACP
- Mayo Clinic
- n/a
- specificity of NACP was tested with immunohistochemistry and western blots of cell lines stably transfected with alpha-synuclein. Two mono-clonal antibodies were also used to confirm the results with NACP in select western blots and immunohistochemistry.
- Gwinn-Hardy et al. Acta Neuropathol 2000; 99: 663-672

Tau E1
- Leonard Petrucelli gift
- In heat-stable preparations from human brains, antibody E-1 bound to multiple bands (Figure 1 A). The antibody E-1-positive proteins migrated in a region corresponding to microtubule-associated protein tau. The staining pattern was comparable to that displayed with Tau-1 (Figure 1A) and other anti-tau antibodies. Preimmune serum did not react with brain proteins. The antibody E-1-reactive proteins from animal brains were not found in the perchloric acid-soluble supernatant. Instead they were detected in the acid precipitates. Absorption of Ab E-1 antiserum with E-1 peptide removed the antibodies responsible for the staining of 60- to 68-kd proteins on immunoblots
- AB_2819185
- Crowe, Am J Pathol, 1991
Tau pS396
- Abcam 109390
- Tested applications Suitable for: Dot blot, IHC-Fr, WB, IP Unsuitable for: ICC/IF Species reactivity Reacts with: Mouse, Rat, Human
- AB_10860822
- https://www.abcam.com/tau-phospho-s396-antibody-epr2731-ab109390.html

RD4
- Millipore
- 05-804
- specificity of RD4 was determined with immunohistochemistry in the following tauopathies: Pick's disease, progressive supranuclear palsy and Alzheimer's disease. Biochemical confirmation of specificity utilized western blots of these three diseases, as well as recombinant tau proteins. RD4 stained inclusions in progressive supranuclear palsy but not in Pick's disease. In Alzheimer's disease, neurofibrillary tangles tangles were immunolabeled with RD4, except for extracellular tangles.
- Togo et al. J Neuropathology and Experimental Neurology, 2002; 61(6):547-556

PHF1
- Peter Davies gift
- Creation and validation of PHF1 tau have been completed.
- Greenberg and Davis. Proc Natl Acad Sci USA 1990; 87(15):5827-31

Human research participants

Policy information about studies involving human research participants

Population characteristics	Total sample size= 32. Average age at death = 71 (range 46-86), 16 women/16 men, 6 APOE e4 carriers
Recruitment	The Neurodegenerative Research Group (NRG), Mayo Clinic, recruited patients who presented to the Department of Neurology with an AOS suspected to be secondary to a degenerative process between 7/1/2010 and 12/31/2020. Only patients over age 18 with an informant to provide independent evaluation of functioning, and who spoke English as their primary language (including bilingual patients), were enrolled; none were 18 or younger. Only patients that have subsequently died and undergone an autopsy were included in the study. Potential study biases include a bias towards who's family chooses to have the patient undergo an autopsy at the time of death. We have not, however, found any demographic differences between those who do and do not get an autopsy in our cohort. Another bias is that we had no minority or hispanic patients in our cohort which may limit generalizability to other races or ethnicities.
Ethics oversight	Mayo Clinic Institutional Review Board

Note that full information on the approval of the study protocol must also be provided in the manuscript.

Clinical data

Policy information about clinical studies

All manuscripts should comply with the ICMJEguidelines for publication of clinical research and a completedCONSORT checklist must be included with all submissions.

Clinical trial registration	NCT01818661; NCT03313011; NCT01623284
Study protocol	The full study protocols are not publicly accessible as this is not mandated by the NIH and this is not a clinical trial. Our protocol is held my the Mayo Clinic Institutional Review Board.
Data collection	Mayo Clinic, Rochester, MN, between 7/1/2010 and 12/31/2020
Outcomes	Outcomes included cross-sectional and longitudinal clinical (neurological, speech and language, neuropsychological) and neuroimaging (MRI, DTI, FDG-PET) measures, as well as genetic (APOE genotype and H1/H2 haplotype), histological (semi-quantitative tau lesion count and neuronal loss), and biochemical (insoluble tau subunits via western blot) measures. These outcome measures were defined at the time of submission of the NIH grant in 2010 based on the aims of our study and our hypotheses at the time of NIH-grant submission. Clinical measures were assessed utilizing multiple validated scales. At each visit a board certified behavioral neurologist, a speech language pathologist and a neuropsychometrist, supervised by a board certified neuropsychologist, administered these standardized scales. MRI and PET scans were also acquired at each visit. At the first visit blood was drawn and sent to the genetic laboratory for genetic screening and genotyping. All data was analyzed using predefined statistical methods outlined in the manuscript. Statistical analyses were conducted by a masters level statistician with some analyses overseen by a PhD level statistician.

Magnetic resonance imaging

Experimental design

Design type	Only structural MRI used in the study - volumes measured on MPRAGE and white matter integrity measured on DTI
Design specifications	N/A
Behavioral performance measures	N/A

Acquisition

Imaging type(s)	Structural and diffusion
Field strength	3T
Sequence & imaging parameters	Sagittal 3D MPRAGE sequence: TR/TE/T1, 2300/3/900 ms; flip angle 8°, 26-cm FOV; 256 x 256 in-plane matrix with a phase FOV of 0.94, slice thickness of 1.2 mm, in-plane resolution 1mm
Area of acquisition	Whole brain

Diffusion MRI ☒ Used ☐ Not used

Parameters	Single-shot echo-planar DTI pulse sequence (TR= 10,200ms; in-plane matrix 128/128; in-plane resolution 2.7, FOV 35cm; phase field of view (FOV) 0.66; 42 diffusion encoding steps and four non-diffusion weighted T2 images; 2.7mm isotropic resolution). Parallel imaging with a sensitivity encoding (SENSE) factor of two

Preprocessing

Preprocessing software	SPM12 (revision 8369) with default settings (https://www.fil.ion.ucl.ac.uk/spm/); Advanced Registration Tools (ANTs) version 1.9.x (https://github.com/ANTsX/ANTs) ; MCALT v1.4 (https://www.nitrc.org/projects/mcalt/)
Normalization	All MPRAGE scans were normalized to the Mayo Clinic Adult Lifespan Template (MCALT) using Advanced Normalization Tools (Avants et al., 2008). The parameters from normalizing the MPRAGE scans to the MCALT template, were used to propagate atlases to native MPRAGE space and used to output regional volumes. Each DTI image (fractional anisotropy and mean diffusivity images) was non-linearly co-registered and normalized to a 1mm isotropic Montreal Neurological Institute (MNI) 152 standard space via the FMRIB58_FA template
Normalization template	MCALT in MNI space; FMRIB58_FA template in MNI space
Noise and artifact removal	MPRAGE scans underwent correction for intensity inhomogeneity. Each DTI scan was denoised, corrected for head motion and eddy current distortion using FSL's eddy program, Gibbs ringing, and then skull stripped. The Rician noise bias was then removed using the noise image from denoising.
Volume censoring	N/A

Statistical modeling & inference

Model type and settings	Multiple regression analyses in SPM12, including age and gender as covariates - to perform voxel-level group comparisons
Effect(s) tested	N/A

Specify type of analysis: ☐ Whole brain ☒ ROI-based ☐ Both

Anatomical location(s)	Regional MRI volumes were calculated using the MCALT atlas. Regional DTI fractional anisotropy and mean diffusivity were calculated using the JHU "Eve" WM atlas
Statistic type for inference (See Eklund et al. 2016)	Voxel-wise analyses for DTI.
Correction	Voxel-level results were assessed corrected for multiple comparisons using the family wise error correction at $p<0.05$ and uncorrected at $p<0.001$, with age and gender included as covariates.

Models & analysis

n/a	Involved in the study
☒	☐ Functional and/or effective connectivity
☒	☐ Graph analysis
☒	☐ Multivariate modeling or predictive analysis
